# Catalytic behavior of metal catalysts in high-temperature RWGS reaction: *In-situ* FT-IR experiments and first-principles calculations

**DOI:** 10.1038/srep41207

**Published:** 2017-01-25

**Authors:** Sungjun Choi, Byoung-In Sang, Jongsup Hong, Kyung Joong Yoon, Ji-Won Son, Jong-Ho Lee, Byung-Kook Kim, Hyoungchul Kim

**Affiliations:** 1 High-Temperature Energy Materials Research Center, Korea Institute of Science and Technology, 5 Hwarang-ro 14-gil, Seongbuk-gu, Seoul 02792, Republic of Korea; 2 Department of Chemical Engineering, Hanyang University, 222 Wangsimni-ro, Seongdong-gu, Seoul 04763, Republic of Korea; 3 Nanomaterial Science and Engineering, Korea University of Science and Technology, KIST Campus, 5 Hwarang-ro 14-gil, Seongbuk-gu, Seoul 02792, Republic of Korea

## Abstract

High-temperature chemical reactions are ubiquitous in (electro) chemical applications designed to meet the growing demands of environmental and energy protection. However, the fundamental understanding and optimization of such reactions are great challenges because they are hampered by the spontaneous, dynamic, and high-temperature conditions. Here, we investigated the roles of metal catalysts (Pd, Ni, Cu, and Ag) in the high-temperature reverse water-gas shift (RWGS) reaction using *in-situ* surface analyses and density functional theory (DFT) calculations. Catalysts were prepared by the deposition-precipitation method with urea hydrolysis and freeze-drying. Most metals show a maximum catalytic activity during the RWGS reaction (reaching the thermodynamic conversion limit) with formate groups as an intermediate adsorbed species, while Ag metal has limited activity with the carbonate species on its surface. According to DFT calculations, such carbonate groups result from the suppressed dissociation and adsorption of hydrogen on the Ag surface, which is in good agreement with the experimental RWGS results.

The demand for carbon-neutral technologies has increased over the last two decades because of the negative effects of global warming and climate change caused by excessive carbon dioxide (CO_2_) emissions^1^,^2^. Various techniques^3^,^4^,^5^ have been studied to directly or indirectly reduce and/or utilize CO_2_ in the atmosphere. Among these approaches, high-temperature chemical reactions have attracted increasing interest as next-generation fuel processing and energy conversion/storage processes^6^,^7^,^8^. The reverse water-gas shift (RWGS) reaction is one of the most promising high-temperature chemical reactions because of its thermodynamic favourability and its direct gas-phase reaction^3^, expressed as


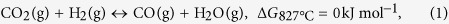


where *G* is the Gibbs free energy. In addition, the RWGS reaction offers further (CO-based) synthetic processes to obtain methanol through CO_2_ hydrogenation^9^, or long-chain hydrocarbons through the Fischer–Tropsch reaction^10^.

To date, various catalysts, including supported noble metals (e.g., Pt/8Y_2_O_3_-ZrO_2_ and Rh/Al_2_O_3_)^11^,^12^, supported transition metals (e.g., Ni/Na/CeO_2_ and Cu/SiO_2_)^13^,^14^,^15^, and perovskite oxide catalysts (e.g., La_1−*x*_Sr_*x*_CoO_3_ and BaCe_*x*_Zr_0.8−*x*_Y_0.16_Zn_0.04_O_3_)^8^,^16^ have been used in the RWGS reaction. Despite the comprehensive studies conducted to develop high-performance catalysts for the RWGS reaction, a fundamental understanding of the roles of metal catalysts and their mechanisms during the spontaneous, dynamic, and high-temperature RWGS reaction is lacking. Hence, *in-situ* measurements and density functional theory (DFT) predictions of the surface adsorption and intermediate functional groups are required, which will substantially contribute to the development of advanced high-temperature RWGS reactions.

In this study, we investigated the RWGS reaction catalysed by four different metal catalysts (Pd, Ni, Cu, and Ag) on an inert Al_2_O_3_ support as a function of temperature (*T*) and H_2_-to-CO_2_ ratio (*R*_mix_), and developed a comprehensive understanding of their roles at high temperatures using *in-situ* Fourier-transform infrared spectroscopy (FT-IR) and DFT calculations. These advanced experimental and computational techniques allow us to directly study the adsorbed functional groups by analysing the IR spectra of the surfaces of the metal catalysts and comparing the results with the DFT-predicted reaction energies and electronic-state changes of the intermediate functional groups.

## Results and Discussion

### Synthesis and characterization of metal catalysts on Al_2_O_3_ support

**Figure 1:**
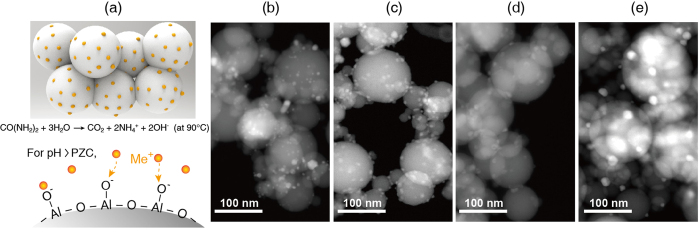
Catalyst synthesis method and synthesized catalyst. (**a**) Schematic representation of the deposition precipitation method with urea hydrolysis, CO(NH_2_)_2_ + 3H_2_O → CO_2_ + 2NH_4_^+^ + 2OH^−^, at 90 °C. High-angle annular dark-field TEM images of the as-synthesized catalysts: (**b**) Pd/Al_2_O_3_, (**c**) Ni/Al_2_O_3_, (**d**) Cu/Al_2_O_3_, and (**e**) Ag/Al_2_O_3_. All scale bars correspond to 100 nm.

**Figure 2:**
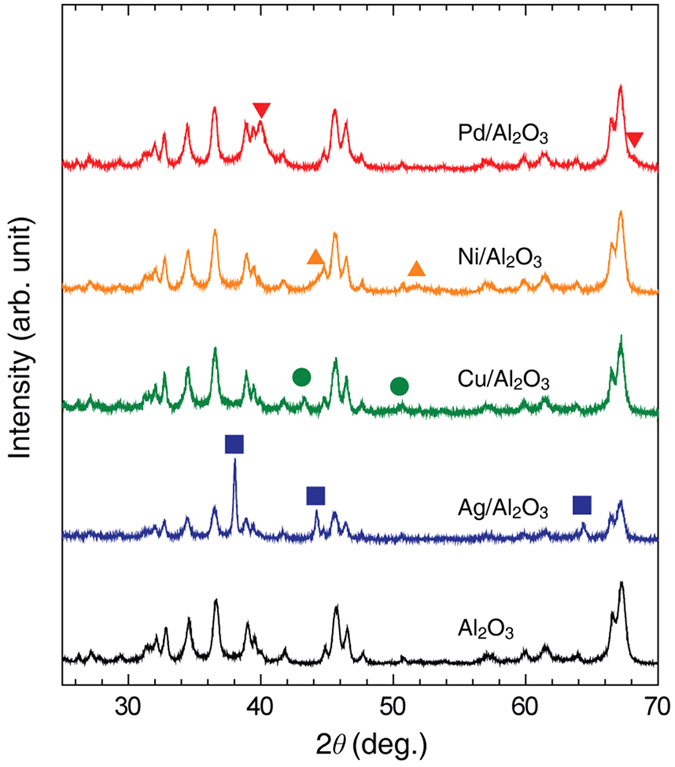
XRD patterns of the catalysts reduced at 600 °C for 2 h. The red, yellow, green, and blue lines and symbols correspond to the supported Pd, Ni, Cu, and Ag catalysts, respectively. The black line represents the XRD pattern of the Al_2_O_3_ support.

To analyse the roles of the metal catalysts in the high-temperature RWGS reaction, we synthesized and examined various metal nanocatalysts supported on Al_2_O_3_ spherical powder. Figure 1(a) presents a schematic illustration of catalyst synthesis by deposition-precipitation with urea hydrolysis. The surface charge of the Al_2_O_3_ powder changes with solution pH, which facilitates positive metal ion adsorption above the point of zero charge (PZC; for Al_2_O_3_, PZC = pH 8.5–9.0^17^). At 90 °C, urea can decompose in water to form hydroxide anions (OH^−^), thereby increasing the pH. We also applied freeze dehydration to avoid the agglomeration and coalescence of nanocatalysts during the drying and calcination step. Finally, various metal nanocatalysts (well-dispersed on an Al_2_O_3_ support) were successfully obtained for application in high-temperature RWGS reactions. Transmission electron microscopy (TEM) images of the reduced metal/Al_2_O_3_ catalysts powder are shown in Fig. 1(b–e). The reduced metal catalysts have an average particle diameter of ~10 nm with standard error of ~0.4, while the spherical Al_2_O_3_ support has a diameter of ~40 nm (see Table S1 in Supplementary Information). Note that the average particle size and its standard error were determined using TEM image processing over 50 randomly selected particles on Al_2_O_3_. We also verified the surface area of the as-prepared metal/Al_2_O_3_ samples using the Brunauer–Emmett–Teller surface area (*S*_BET_). All samples exhibited similar surface areas (*S*_BET_ values) in the range of 29.0–34.2 m^2^ g^−1^ (see Table S1 in Supplementary Information). The reducibility of the as-synthesized metal catalysts was examined by temperature-programmed reduction (TPR). The profiles in Fig. S1 (Supplementary Information) noticeably show their inherent reducing features [for Pd, decomposition of palladium hydrides at ~80 °C^18^; for Ni, strong interaction of Ni(O)-Al_2_O_3_ and its reduction at ~500 °C^19^; for Cu, the reduction of surface and bulk CuO at ~200 °C^20^; for Ag, no oxidation under this calcination condition]. The X-ray diffraction (XRD) patterns shown in Fig. 2 confirmed the crystalline structures of the metal nanocatalysts. Ignoring the complex XRD spectra of the Al_2_O_3_ support, we identified the metallic state of each catalyst. Using such fully-reduced metal catalysts (Pd, Ni, Cu, and Ag), we investigated the RWGS performance as a function of reactant mixing ratio and temperature using a gas chromatograph (GC) and *in-situ* FT-IR experiments.

### RWGS performance of various metal catalysts

**Figure 3:**
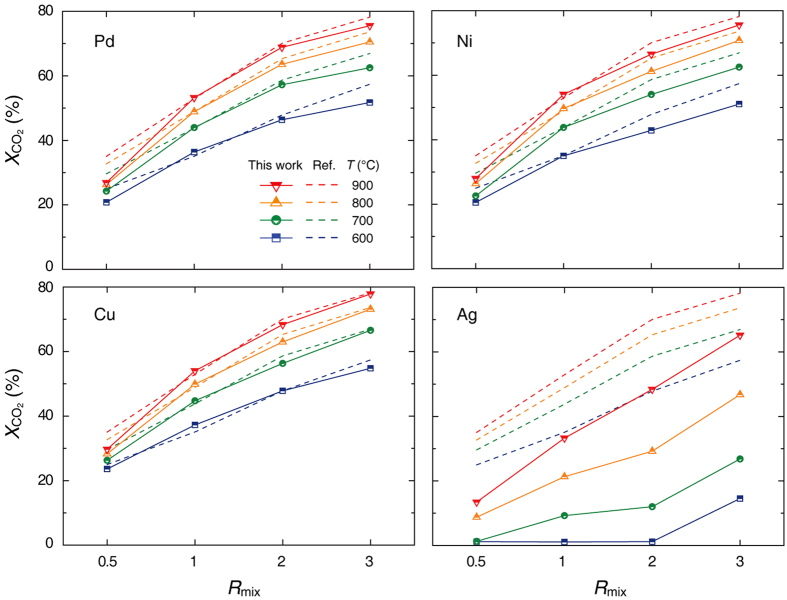
CO_2_ conversion of each catalyst with respect to temperature and *R*_mix_. The red, yellow, green, and blue lines correspond to temperatures of 900, 800, 700, and 600 °C, respectively. For reference, all *X*co_2_ profiles of the empty tube and Al_2_O_3_ support are presented in Fig. S2 (Supplementary Information).

**Figure 4:**
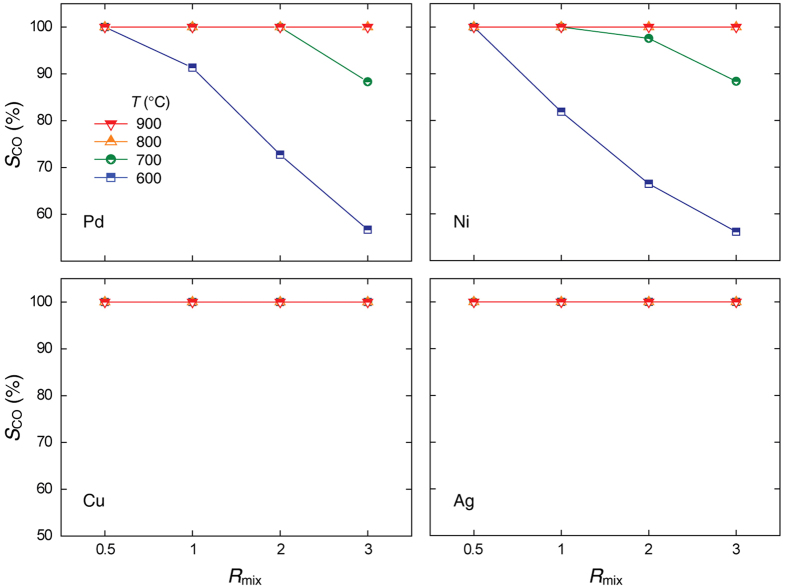
CO selectivity of each catalyst with respect to temperature and *R*_mix_. The red, yellow, green, and blue lines correspond to temperatures of 900, 800, 700, and 600 °C, respectively. For reference, the *S*co of the Al_2_O_3_ support in all cases is 100% in spite of its limited *X*co_2_ performance.

Figures 3 and 4 present the RWGS catalytic performance, CO_2_ conversion (*X*co_2_), and CO selectivity (*S*co) [see Eqs (2) and (3) in Methods] achieved by various metal catalysts on the Al_2_O_3_ support. In general, the RWGS reaction is considered an equilibrium gas-phase process without any dynamic or transient conditions^3^. By formulating thermodynamic models for the RWGS reaction^21^, the equilibrium compositions of product gases can be predicted for various temperatures and reactant gas ratios at a specific pressure. The thermodynamic equilibrium performance of *X*co_2_ under our experimental conditions is presented as a dashed line in Fig. 3. In addition, the preliminary experiments using the Al_2_O_3_ support alone and a blank reactor were summarized in Fig. S2 (Supplementary Information). These preliminary results—associated with the possible catalytic effects of the Al_2_O_3_ support (at 800 °C, *X*co_2_ ≈ 15.9 and 27.9% for *R*_mix_ = 1 and 3) and blank system (at 800 °C, *X*co_2_ ≈ 0.0 and 0.0% for *R*_mix_ = 1 and 3)—also serve as a reference for measuring the RWGS performance of the metal catalysts. These results are in general agreement with the literature^12^, the *X*co_2_ of Al_2_O_3_ ≈ 40% at 800 °C for *R*_mix_ = 4. We expect such conversion results of the Al_2_O_3_ support are caused by the inherent features of the RWGS reaction (gas-phase equilibrium limited and favoured at high temperature)^12^. Interestingly, we observed limited (or suppressed) RWGS reaction in our fixed-bed column reactor system. These results raise questions about the roles of the metal catalysts during the spontaneous, dynamic, and high-temperature RWGS reaction.

As shown in Fig. 3, all metal catalysts except Ag exhibited similar behaviours and maximum conversion activities (reaching the thermodynamic equilibrium limit): at 800 °C, *X*co_2_ ≈ 49.5 and 71.5% for *R*_mix_ = 1 and 3, respectively. In contrast, Ag metal has a significantly lower catalytic activity under all conditions of temperature and *R*_mix_: at 800 °C, *X*co_2_ ≈ 21.3 and 46.7% for *R*_mix_ = 1 and 3, respectively. Surprisingly, these values obtained for Ag metal are approximately 50% lower than those for other metals and the thermodynamic equilibrium conversion. These catalytic RWGS performances of the metal/Al_2_O_3_ catalyst are competitive with Fe_2_O_3_ powder, one of the widely used industrial catalysts for WGS (at *T* = 350 to 450 °C)^22^. As shown in Fig. S3 (Supplementary Information), the overall *X*co_2_ and *S*_CO_ of Fe_2_O_3_ generally increase with temperature, but the measured *X*co_2_ results are slightly scattered and suppressed (at 800 °C, *X*co_2_ ≈ 23.4 and 40.4% for *R*_mix_ = 1 and 3) due to its significant sintering behaviour in a highly reducing atmosphere at high temperature.

Figure 4 presents the *S*co values measured for various metal catalysts at different temperatures and *R*_mix_ values. From the theoretical thermodynamic models^21^ and previous experimental results^23^ for the RWGS reaction, CH_4_ formation is generally observed in the intermediate temperature range (*T* ≤ 700 °C) and/or high *R*_mix_ condition, not above 800 °C. Thus, we found that the *S*co of all catalysts for *T* ≥ 800 °C is 100%. For *T* ≤ 700 °C, however, the catalytic behaviour of CO_2_ conversion was noticeably changed. The *S*co values of the Pd and Ni catalysts decreased with increasing *R*_mix_ due to the formation of CH_4_ at 600–700 °C. In contrast, the Cu and Ag catalysts achieved *S*co values of 100% under all experimental conditions, and no CH_4_ production was observed. Cu and Ag favoured CO production, while Pd and Ni were partially active toward CH_4_ formation. Such a difference was mainly caused by the opposed ability toward H_2_ dissociation of each metal catalyst. The Pd and Ni catalysts including general noble metals (e.g., Pt, Rh, Ru) have higher activity toward H_2_ dissociation than the others, and provide an interesting result of CH_4_ formation at *T* ≤ 700 °C^23^. The details regarding the different abilities of H_2_ dissociation will be discussed later.

Based on the *X*co_2_ and *S*co results in Figs 3 and 4, we found that temperature and the amount of hydrogen (or H_2_ dissociation process) are the dominant factors in high-temperature RWGS reactions, as the Gibbs free energy is negative above 827 °C and CO_2_ gas was stable even at high temperatures in the absence of a reducing atmosphere or reduction process. Additionally, we concluded that the catalytic effects of most metal catalysts (Pd, Ni, and Cu) are rather high, whereas that of Ag metal is generally negligible.

### *In-situ* characterization of surface transient species

**Figure 5:**
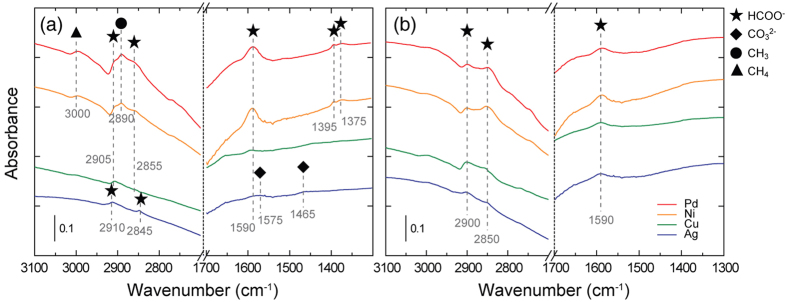
DRIFTS spectra of the RWGS reaction at (**a**) 600 and (**b**) 800 °C with *R*_mix_ = 1. The red, yellow, green, and blue lines correspond to the supported Pd, Ni, Cu, and Ag catalysts, respectively. The star, diamond, circle, and triangle symbols represent intermediate materials of formate, carbonate, adsorbed methyl groups, and methane gas, respectively.

To clarify the roles of metal catalysts in the high-temperature RWGS reaction, we conducted *in-situ* diffuse reflectance infrared Fourier transform spectroscopic (DRIFTS) characterization to identify the intermediate species adsorbed on the metal surface. Figure 5 depicts the DRIFTS spectra of various metal catalysts during the RWGS reaction at 600 and 800 °C. As reported^20^,^24^,^25^, three different formate bands can be assigned based on several vibrational modes. The first bands, *ν*(O-C-O)_as_ and *ν*(C-H), are calculated to be in the regions of 1590 and 2855–2850 cm^−1^, respectively. A second combination band, *ν*(O-C-O)_as_ and *δ*(C-H), is found from 2905 to 2900 cm^−1^. The third formate band, *δ*(C-H) and *ν*(O-C-O)_s_, is located from 1395 to 1375 cm^−1^. For the highly active metal catalysts (e.g., Pd, Ni, and Cu), formate (HCOO^−^) groups were clearly detected as a major adsorbed species in the RWGS reaction. Additionally, methane (CH_4_), adsorbed methyl (CH_3_), and carbonate (CO_3_^2−^) groups were observed in small quantities on the catalyst surface. Gaseous CH_4_ and adsorbed CH_3_ were only found on the Pd and Ni/Al_2_O_3_ catalysts at 600 °C, as indicated by the peaks at 3000 and 2890 cm^−1^ 26,27,28, respectively. This result is in agreement with the suppressed CO selectivity observed at 600 °C due to CH_4_ formation. In contrast, the Ag/Al_2_O_3_ catalyst had two distinct bands not observed in the other highly active metal catalysts: one indicating monodentate carbonate, *ν*(O-C-O)_as_, at 1465 cm^−1^ and one indicating a chelating bidentate carbonate, *ν*(C = O), at 1575 cm^−1^ 25,29,30. We conclude that the presence of these carbonate species is closely related to the lower catalytic activity of the Ag metal catalyst, as formate species are generally formed by the interaction between the spillover of a H atom to an adsorbed CO_2_. Based on literature reports^31^, the catalytic RWGS reaction mostly occurs at the interfacial area between the metal and the support. Considering the limited H_2_ dissociative adsorption from the Al_2_O_3_ support, we cannot expect a sufficient combination of CO_2_ adsorption on the metal and H_2_ dissociative adsorption on the support, especially in the case of Ag, which has a limited capability for H_2_ dissociative adsorption. Therefore, we attribute the superior (or suppressed) CO_2_ conversion with the existence (or absence) of carbonate species resulting from the abundance (or lack) of activated H atoms, as supported by the DFT calculations described in the following section.

### DFT prediction of surface reactions on various metal catalysts

**Figure 6:**
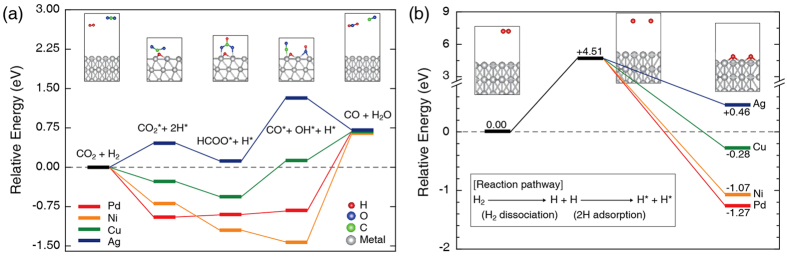
Calculated reaction energy diagram of various metal (111) surfaces. (**a**) Overall energy levels of different metal catalysts along the RWGS reaction pathway. (**b**) Energy diagram of H_2_ dissociation and adsorption. The red, yellow, green, and blue lines correspond to the Pd, Ni, Cu, and Ag surfaces, respectively. The inset image provides a representative illustration of each reaction step.

To predict the reaction mechanisms on the metal surfaces, we considered the overall reaction pathway, and observed their behaviours with relative energies using the DFT formalism. Here, we adopted the slab model (first three layers free to relax) to simulate the metal catalysts, considering the computational convenience and cost for metal nanocatalysts (~10 nm diameter and highly-crystalline structure). Note that some reaction energies such as H_2_ dissociative chemisorption may lead to small uncertainties due to the structural limits and coverage effects of the slab model. First, the surface energy results of various metals were investigated and are summarized in Table 1. Among the four metals, Ag has the lowest surface energy and Ni the highest. The (111) surface has the lowest energy among the three surfaces in the studied metals, followed by the (100) surface and then the (110) surface, except in the case of Ag. We then calculated the reaction energy diagram on various metal (111) surfaces to simulate the overall RWGS pathway including a formate intermediate and a dissociative H adsorbent, which are experimentally observed in above *in-situ* characterization. Figure 6(a) presents the overall energetics of different metal catalysts for each reaction pathway, a total of 5 elementary reaction steps (including 3 intermediate adsorbed states): (1) H_2_(g) + CO_2_(g) ↔ (2) CO_2_^*^ + 2H^*^ ↔ (3) HCOO^*^ + H^*^ ↔ (4) CO^*^ + OH^*^ + H^*^ ↔ (5) CO(g) + H_2_O(g), where the asterisks (*) denote adsorbed species. Most reaction energies are in general agreement with the previous (R)WGS results^32^,^33^,^34^, favourable CO_2_ hydrogenation, and (temperature-dependent endothermic) limited process. Considering the difficulty of adsorbing a non-polar linear CO_2_ molecule, surprisingly, all three metals except Ag showed favourable energetics in the reaction step (1) to (2). In addition, the unfavourable CO_2_ hydrogenation of the Ag catalyst continues to the other reaction steps including the formation of the formate intermediate [the reaction step (2) to (3)] and adsorbed carbon monoxide [the reaction step (3) to (4)]. In order to clarify such a different and limited reaction nature of Ag metal in the RWGS process, we calculated the energies for the surface hydrogen dissociation and adsorption energies on the metals studied. Among the H adsorption energies calculated on the (111) surfaces, the Ag (111) surface had the lowest value, which is less than the H_2_ dissociation energy (H_2_ → 2H). According to the general H_2_ reaction mechanism of RWGS on a metal surface^35^, we considered two reaction steps, (i) H_2_ ↔ 2H for H_2_ dissociation and (ii) 2H ↔ 2H^*^ for H adsorption. The relative energy diagrams for H_2_ dissociation and adsorption on various metal (111) surfaces are depicted in Fig. 6(b), showing that only the Ag surface is unfavourable for H adsorption. This observation is highly consistent with the general understanding in the literature^36^ and our experimental findings mentioned above. The H-coverage effects (0.11 to 1 ML) on the metal surfaces are predicted to be negligible (see Table S2 in Supplementary Information). Considering our experimental conditions (0.5 ≤ *R*_mix_ ≤ 3), we clarify that our DFT analyses and results remain valid even under conditions of high H_2_ concentration. Thus, the dissociative adsorption of H_2_ on the Ag surface cannot be successfully promoted because the initial gas state (H_2_) is more stable than the dissociative adsorption state (H). In contrast, the Pd, Ni, and Cu catalysts have higher H adsorption energies and easily produce H-adsorbed species. Thus, these catalysts can be used to promote the RWGS reaction.

**Figure 7:**
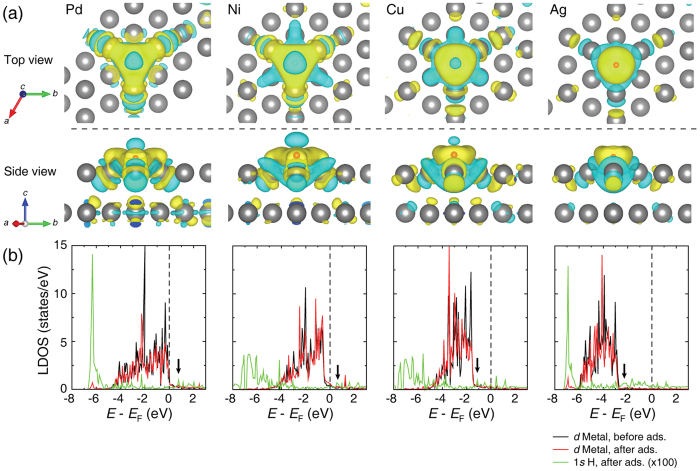
Electronic states calculation of each metal catalyst in H_2_ dissociation and adsorption. (**a**) Charge density difference for each metal with an adsorbed H atom. The isosurface level is 0.0067 *e* Å^−3^. The orange sphere is an adsorbed H atom. The yellow and cyan surfaces indicate electron density accumulation and depletion, respectively. (**b**) LDOS changes for the metal catalysts with an adsorbed H atom. The black and red lines correspond to metal *d*-orbitals before and after adsorption, respectively. The green line represents the 1 *s* orbital of an adsorbed H atom multiplied by a factor of 100. The dashed line is the Fermi level (*E*_F_) and the black arrow is anti-bonding states of H 1*s*-metal 3*d* (or 4*d*).

Analyses of the ionic charge and spatial charge distribution are useful for understanding the surface adsorption of H atoms onto a metal surface. Figure 7(a) shows the charge density difference for H adsorbed on various metal surfaces (Pd, Ni, Cu, and Ag) and the density difference surfaces at an iso-value of 0.0067 *e* Å^−3^. According to the charge density difference, the adsorbed H atom is generally polarized, generating a dipole that interacts with the point charge of a metal atom. This description is in general agreement with the findings for Pd, Ni, and Cu. Additionally, H adsorption on the Pd, Ni, and Cu surfaces affects their charge densities down to the second subsurface layer. However, the Ag surface with an adsorbed H atom suppresses the charge polarization and does not extensively interact electrostatically with surface silver atoms. The suppressed polarization of an adsorbed H atom results in the limited surface catalytic reaction of formate intermediate surface- and gas-phase species. To verify the charge distortion mentioned above, we investigated the change of electronic local density-of-states (LDOS) of H adsorbed on various metal surfaces. As shown in Fig. 7(b), the anti-bonding resonances between 1 *s* of H and 3*d* (or 4*d*) of the metal layer were observed above the *d*-bands after adsorption. Similar to the literature^37^, the anti-bonding states of Pd and Ni are empty (located above the Fermi level, *E*_F_), while those of Cu and Ag are filled with electrons (located below the *E*_F_). This indicates the repulsive interactions of the H and metal (Cu and Ag) atom likely result in the unstable bond behaviour. The distance from the *E*_F_ to the centre of the *d*-band is highly related to the local reactivity of the *d*-band^38^. From Fig. 7(b), the lowest *d*-band centre (i.e., higher *E*_*d*-band,c_ − *E*_F_) of Ag metal results in the weakest bonding and the lowest reactivity. We conclude that the metal surfaces studied, except Ag, encourage H dissociation and a significant charge polarization of adsorbed H atoms, facilitating the formation of specific intermediate formate functional groups (e.g., HCOO^−^) that promote the RWGS reaction. These findings agree well with the above-mentioned GC experimental results and *in-situ* FT-IR data.

## Conclusion

In summary, the catalytic activity and adsorption properties of various metals (Pd, Ni, Cu, and Ag) on an Al_2_O_3_ support for the high-temperature RWGS reaction were investigated by *in-situ* FT-IR experiments and DFT calculations. The CO_2_ conversion shows the maximum activity on the Pd, Ni, and Cu catalysts, reaching the thermodynamic limit, while that of Ag metal is suppressed. According to our *in-situ* high-temperature FT-IR measurements and comprehensive DFT calculations, we directly observed that the formate group is an intermediate adsorbed species on all highly activated catalysts during the high-temperature RWGS reaction. In addition, this formation of a formate group is noticeably determined by the H_2_ dissociation and H adsorption capabilities of the metal surface. Interestingly, the suppressed catalytic activity of Ag is clearly verified by the reaction energy diagram and *d*-band analysis. Such an understanding of the roles of metal catalysts and their mechanisms during the high-temperature RWGS reaction allows for technological advances for superior high-temperature RWGS catalysts.

## Methods

### Catalyst preparation and characterization

Ag(NO_3_)·6H_2_O (Sigma-Aldrich), Cu(NO_3_)_2_·2.5H_2_O (Sigma-Aldrich), Pd(NO_3_)_2_·2H_2_O (Sigma-Aldrich), and Ni(NO_3_)_2_·6H_2_O (Sigma-Aldrich) were used as the metal precursors. To suppress any catalytic or size effects originating from the support material, spherical Al_2_O_3_ powder (Alfa Aesar, specific surface area = 28 m^2^ g^−1^) was chosen as the support. To prepare the catalyst, Al_2_O_3_ was dispersed in water with vigorous stirring. An appropriate amount of the metal precursor (5 wt%) was dissolved in water. Next, urea (Yakuri Pure Chemical) and the metal precursor were poured into the Al_2_O_3_ slurry, and this mixed solution was heated to 90 °C with vigorous stirring. After 4 h of reaction, the mixed solution was rapidly frozen using liquid nitrogen and dried in a freeze-dryer (FD 5512, Ilshin) overnight. The dried powder was then calcined in an electric box furnace for 3 h at 400 °C.

The *S*_BET_ of the reduced catalysts were determined using the multipoint BET measurement (Quadrasorb SI, Quantachrome) with N_2_ adsorption, as summarized in Table S1 (Supplementary Information). Prior to BET measurement, the catalyst samples were degassed in vacuum at 130 °C for 12 h. The TPR spectra (AutoChem II 2920, Micromeritics) of the as-prepared powders were obtained by using 10 mol% H_2_ balanced in Ar with a flow rate of 50 mL min^−1^ (*T* = 50 to 900 °C, ramp rate = 10 °C min^−1^). All powders were pre-oxidized at 400 °C for 1 h before the TPR measurement.

The crystalline phases of the reduced catalysts were examined using XRD (D/Max-2500, Rigaku) with Cu-K_*α*_ radiation. All XRD patterns were obtained in the 2*θ* range of 20 to 80° with a step size of 0.01°. The size and morphology of the metal catalysts on the Al_2_O_3_ powder were observed by TEM (Talos F200X, FEI) operated at 200 kV. TEM specimens were prepared by dispersing the catalyst powder in ethanol and then placing the sample onto a porous carbon film supported on a Cu grid (or a nickel grid in the case of Cu metal).

### Gas chromatography and IR measurements

The RWGS reaction was conducted in a fixed-bed tubular quartz reactor operated at atmospheric pressure. The synthesized catalyst powder (0.3 g) was placed in the middle of the reactor, on a porous quartz bed fixed by blowtorching. The reactor was placed inside a tube furnace to control the reaction temperature using a proportional-integral-derivative temperature controller. The reactant gas, which contained H_2_ and CO_2_ with a balance of Ar (total feed flow rate = 100 mL min^−1^), was pre-mixed and fed at *R*_mix_ varying from 0.5 to 3. To prevent surface oxidation of the metallic catalysts, all catalysts were reduced under 10 mol% H_2_ gas balanced in Ar at 600 °C for 2 h before the RWGS reaction. The catalytic activity tests were performed in the temperature range 600 to 900 °C using a GC (7890B, Agilent) with a cold trap to separate steam from the product gas. Two columns with different reference flows (Ar and He) were used to maximize the thermal conductivity signals of the product gases. The catalytic activity was evaluated by the CO_2_ conversion and CO selectivity, as given by


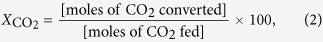



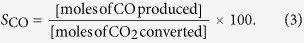


We used *in-situ* DRIFTS to investigate the materials adsorbed on the catalysts during the RWGS reaction. The DRIFTS system was composed of an FT-IR spectrometer (Nicolet 6700, Thermo Scientific) and a high-temperature reaction chamber (DiffusIR, PIKE Technologies). All IR spectra were recorded using a HgCdTe detector over the wavenumber range 4000 to 650 cm^−1^ at a 4 cm^−1^ resolution, and 128 scans were collected at each wavenumber step. All metal catalysts were reduced at 600 °C prior to use in the *in-situ* IR experiments, and ~7 mg catalyst powder was loaded in a porous Al_2_O_3_ crucible serving as a reactor in a high-temperature gas flow system. The sample was then cleaned with high-purity He gas (to remove residual air) for 5 min, and the pre-mixed reactant gas with *R*_mix_ = 1 was introduced into the chamber with increasing temperature.

### First-principles calculations

All calculated energies in this work were obtained using the plane-wave DFT calculations as implemented in the Vienna *ab initio* simulation package^39^. The generalized gradient approximation using Perdew-Burke-Ernzerhof parameterization was employed for the exchange-correlation energy^40^, and the projector augmented wave method^41^,^42^ for describing ionic cores was used. The kinetic energy cutoff for the plane wave basis set was chosen as 1.3 times the energy maximum listed in the potential file of the element. Surface energy and H adsorption energy calculations were performed on slabs consisting of 6 layers of 54 atoms for the (100) and (111) surfaces and 8 layers of 72 atoms for the (110) surface. The layers in the top halves of the slabs were allowed to relax, while the rest were kept at a fixed position with a 15 Å vacuum space (in the *z*-direction). The 2 × 2 × 1 *k*-point grids in the Brillouin zone were sampled by a gamma-centered grid. The surface energy (*E*_surf_) and H adsorption energy (*E*_H,ads_) including the coverage effect were defined as


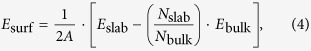



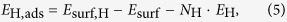


where *E*_*x*_ is the total energy of system *x, N*_*x*_ is the number of atoms in system *x*, and *A* is the area of each surface of the slab. The charge density difference (Δ*ρ*) was also calculated to understand the adsorption behavior between the metal surface and H atom. Δ*ρ* was given by


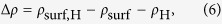


where the subscripts surf, H; surf; and H refer to the charge density of the surface with a H atom, the surface alone, and a free H atom, respectively. To calculate the H_2_ bond-dissociation energy, a rectangular cell with a 20 Å vacuum space in the *x*-, *y*-, and *z*-direction was used for the relaxation of the hydrogen atom or molecule. The calculated bond-dissociation energy for H_2_ is 4.51 eV, in good agreement with the experimental value of 4.48 eV at 0 K^43^.

## Additional Information

**How to cite this article**: Choi, S. *et al*. Catalytic behavior of metal catalysts in high-temperature RWGS reaction: *In-situ* FT-IR experiments and first-principles calculations. *Sci. Rep.* **7**, 41207; doi: 10.1038/srep41207 (2017).

**Publisher's note:** Springer Nature remains neutral with regard to jurisdictional claims in published maps and institutional affiliations.

## Supplementary Material

Supplementary Information

## Figures and Tables

**Table 1:** Comparison of the surface energies of the studied metals.

	Plane	Pd	Ni	Cu	Ag
Surface energy (eV nm^−2^)	(100)	9.66	13.83	9.97	5.85
	(110)	9.84	14.55	10.17	5.73
	(111)	8.53	11.93	9.36	5.57
