# Supplementary material for: Catalytic behavior of metal catalysts in high-temperature RWGS reaction: In-situ FT-IR experiments and first-principles calculations
Source: Sci Rep. 2017 Jan 25;7:41207. doi: 10.1038/srep41207 (PMC5264613; doi:10.1038/srep41207)
Supplement: Supplementary Information [file srep41207-s1.pdf]

[Scientific Reports]

Supplementary Information

Catalytic Behavior of Metal Catalysts in High-Temperature RWGS Reaction: *In-Situ* FT-IR Experiments and First-Principles Calculations

Sungjun Choi[1,2], Byoung-In Sang[2], Jongsup Hong[1], Kyung Joong Yoon[1,3], Ji-Won Son[1,3], Jong-Ho Lee[1,3], Byung-Kook Kim[1] and Hyoungchul Kim[1,3,*]

[1] *High-Temperature Energy Materials Research Center, Korea Institute of Science and Technology, 5 Hwarangno 14-gil, Seongbuk-gu, Seoul 02792, Republic of Korea*

[2] *Department of Chemical Engineering, Hanyang University, 222 Wangsimni-ro, Seongdong-gu, Seoul 04763, Republic of Korea*

[3] *Nanomaterial Science and Engineering, Korea University of Science and Technology, KIST Campus, 5 Hwarangno 14-gil, Seongbuk-gu, Seoul 02792, Republic of Korea*

[*]Corresponding author. E-mail address: hyoungchul@kist.re.kr (H. Kim)

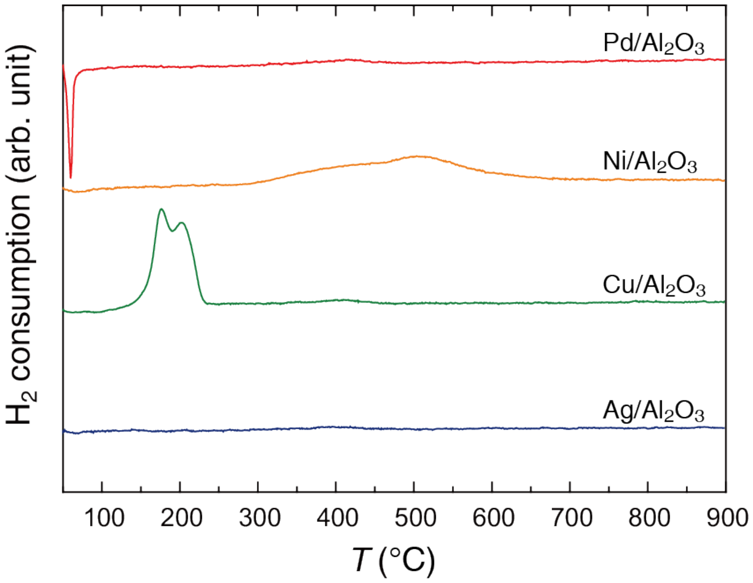

Figure S1. H_2-TPR profiles of as-prepared Metal/Al_2O_3 (Metal = Pd, Ni, Cu, and Ag). The red, yellow, green, and blue lines correspond to the supported Pd, Ni, Cu, and Ag catalysts, respectively. All powders were pre-oxidized at 400 °C for 1 h before the TPR measurement.

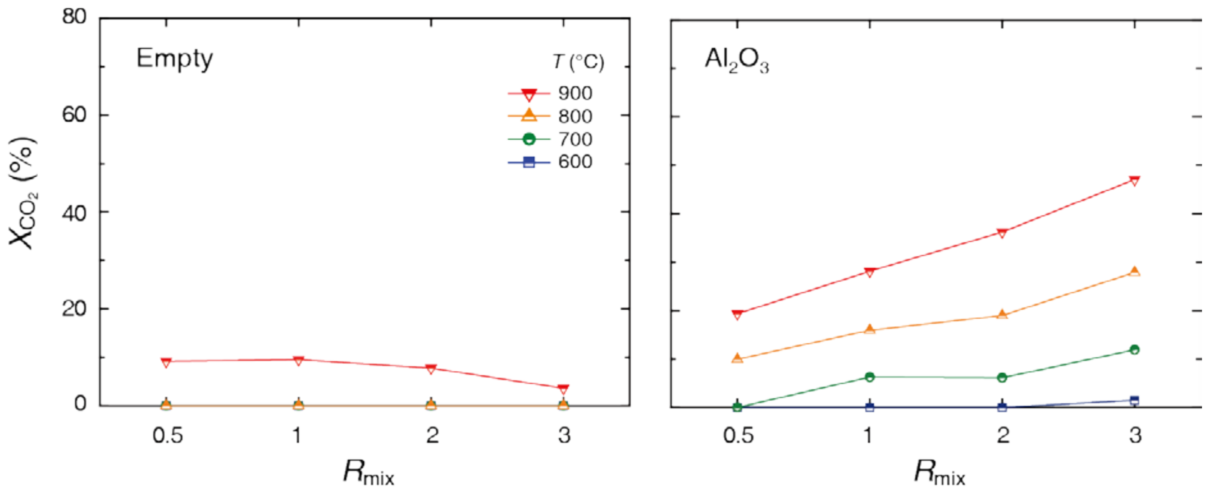

Figure S2. **CO_2 conversion of the empty reactor and Al_2O_3 support with respect to temperature and R_{mix}.** The red, yellow, green, and blue lines correspond to temperatures of 900, 800, 700, and 600 °C, respectively. For your reference, all experiments of empty tube and Al_2O_3 support show the S_{CO} of 100%.

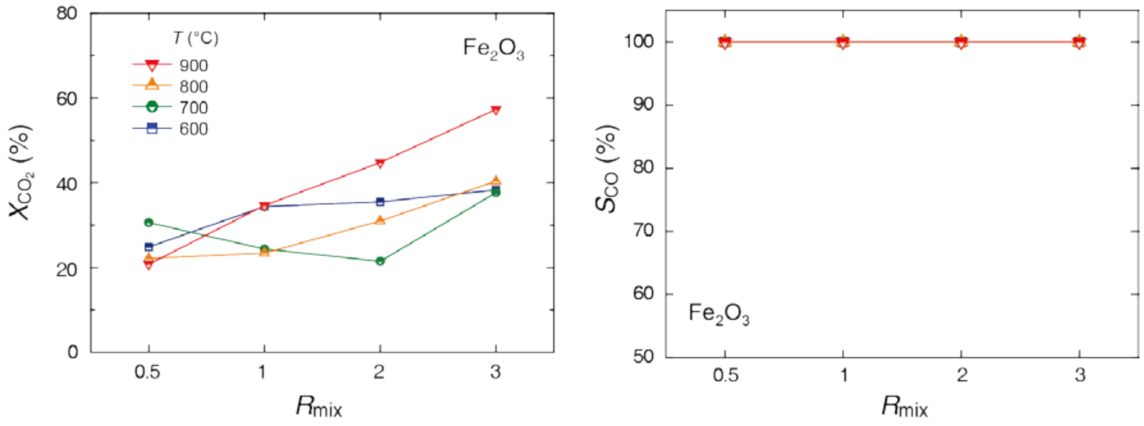

Figure S3. CO_2 conversion of Fe_2O_3 powder with respect to temperature and R_{mix}. The red, yellow, green, and blue lines correspond to temperatures of 900, 800, 700, and 600 °C, respectively. The RWGS reaction was measured with the commercial Fe_2O_3 powder (Alfa Aesar, specific surface area = 30 ~ 60 m^2 g^{-1}) of 0.3 g. All other experimental conditions are same with the Metal/Al_2O_3 samples. The scattered data result from the significant sintering behavior of Fe_3O_4 (during the measurement) in a highly reducing atmosphere at high-temperature.

Metal	d_p (nm)	S_{BET} (m^2 g^{-1})
Pd	8.0	34.2
Ni	8.5	30.7
Cu	10.9	29.4
Ag	11.1	29.0

Table S1. The measured particle diameter (*d_p*) and BET surface areas (*S_{BET}*) of Metal/Al_2O_3 catalysts. The average particle diameters of Pd, Ni, Cu, and Ag catalysts had the standard error of 0.35, 0.45, 0.41, and 0.69, respectively.

Adsorption	$E_{H,ads}$ (eV)			
	Pd	Ni	Cu	Ag
H (0.11 ML)	-2.91	-2.80	-2.41	-2.02
3H (0.33 ML)	-2.88	-2.81	-2.41	-2.03
6H (0.67 ML)	-2.87	-2.81	-2.39	-2.02
9H (1 ML)	-2.85	-2.81	-2.40	-2.03

Table S2. The predicted H adsorption energy on various metal surfaces as a function of H-coverage.
